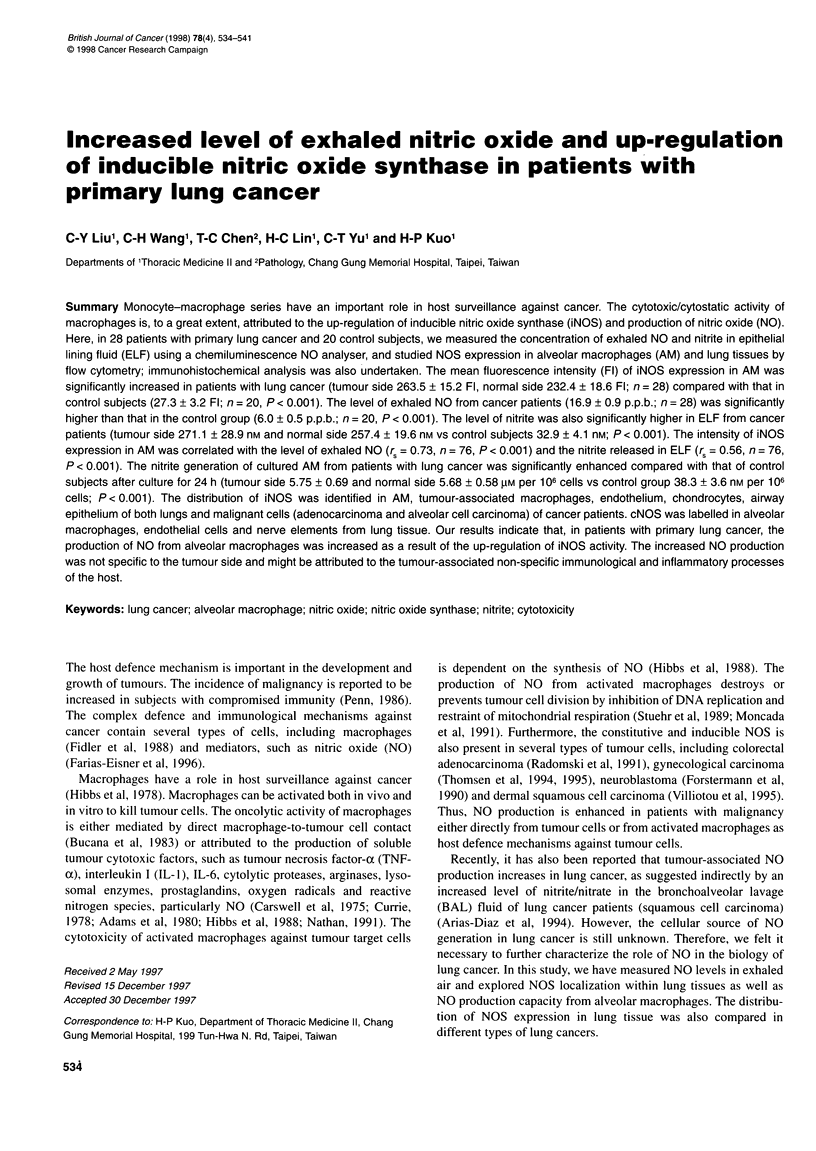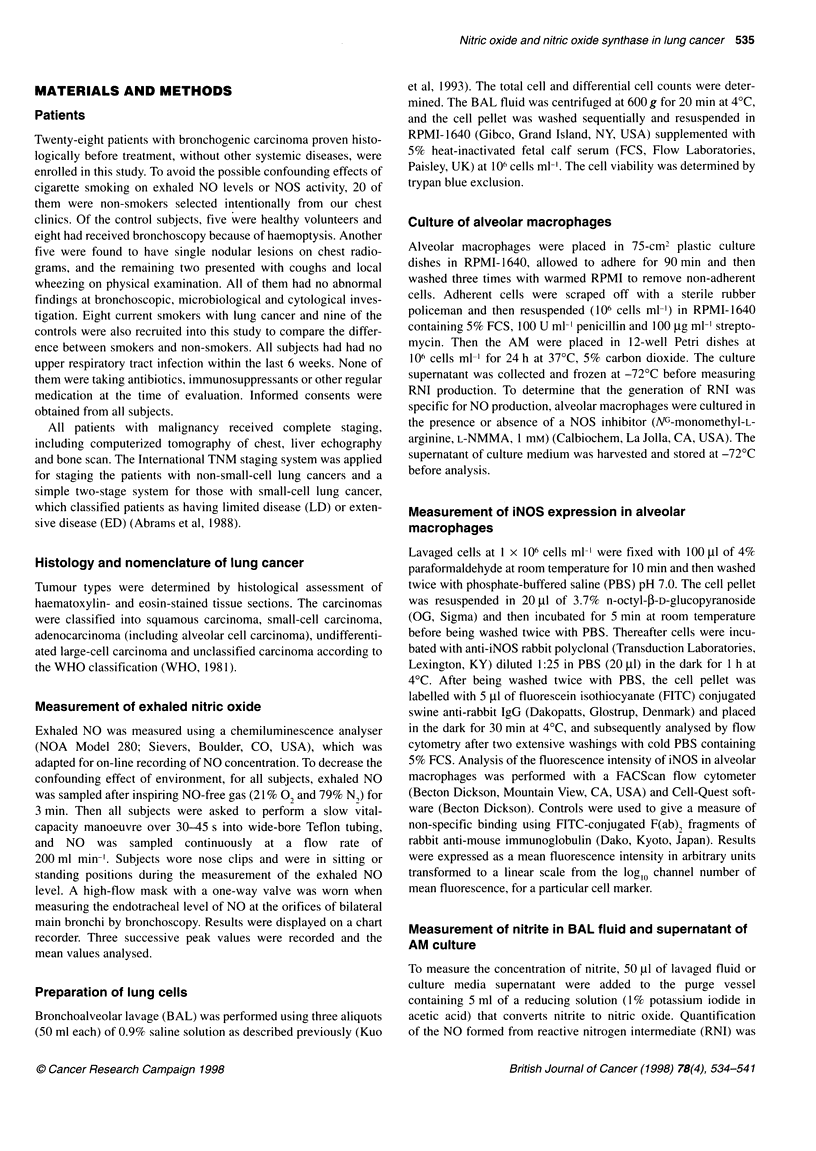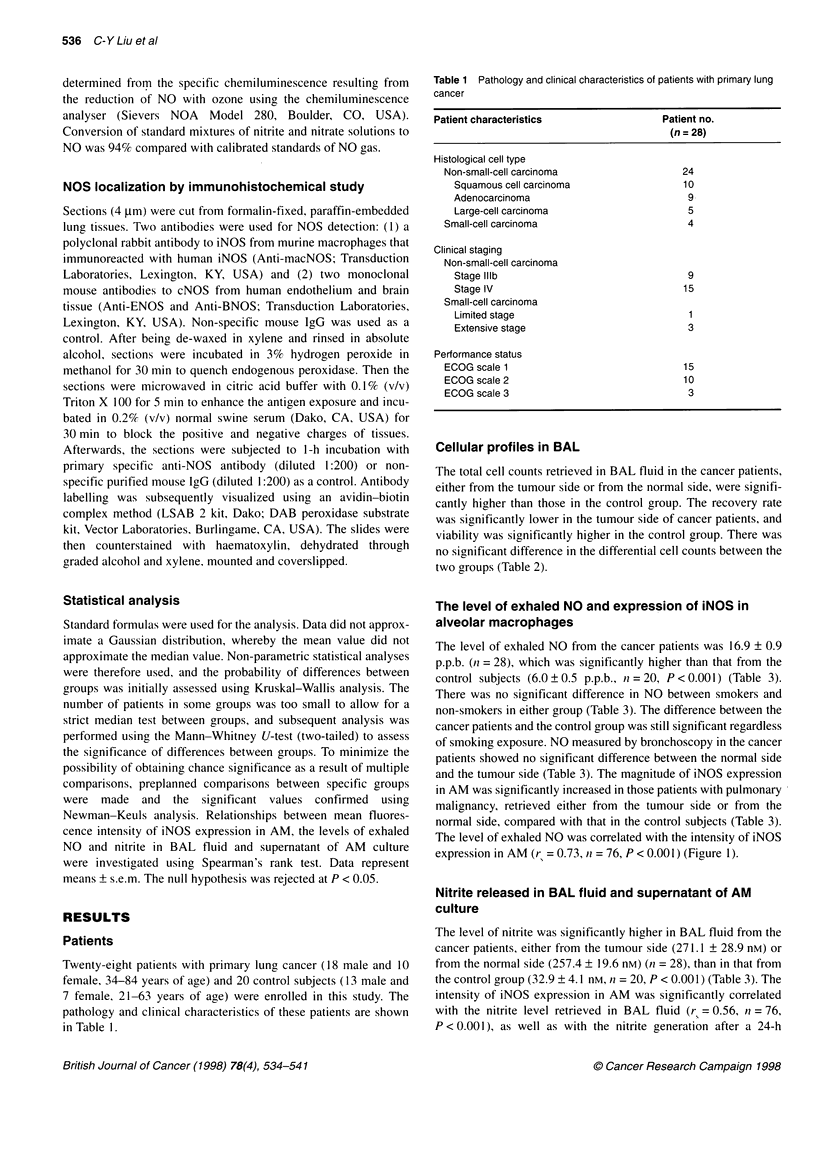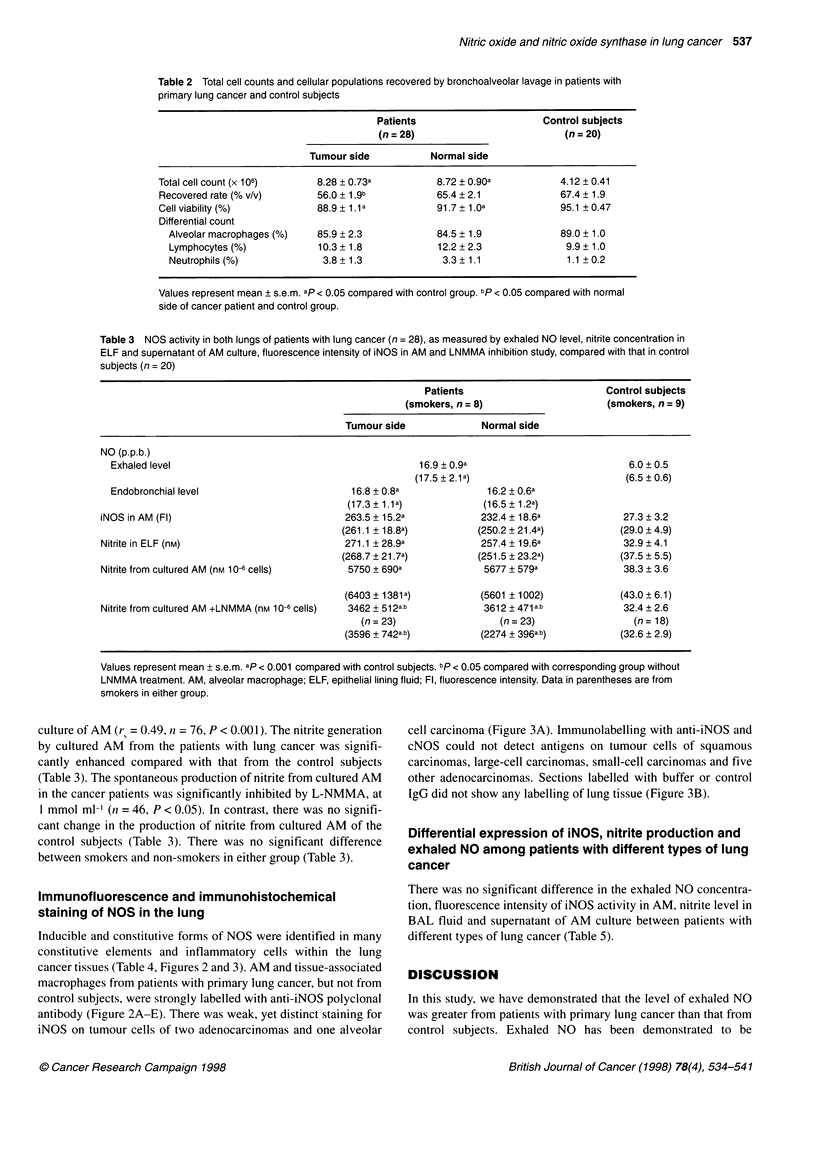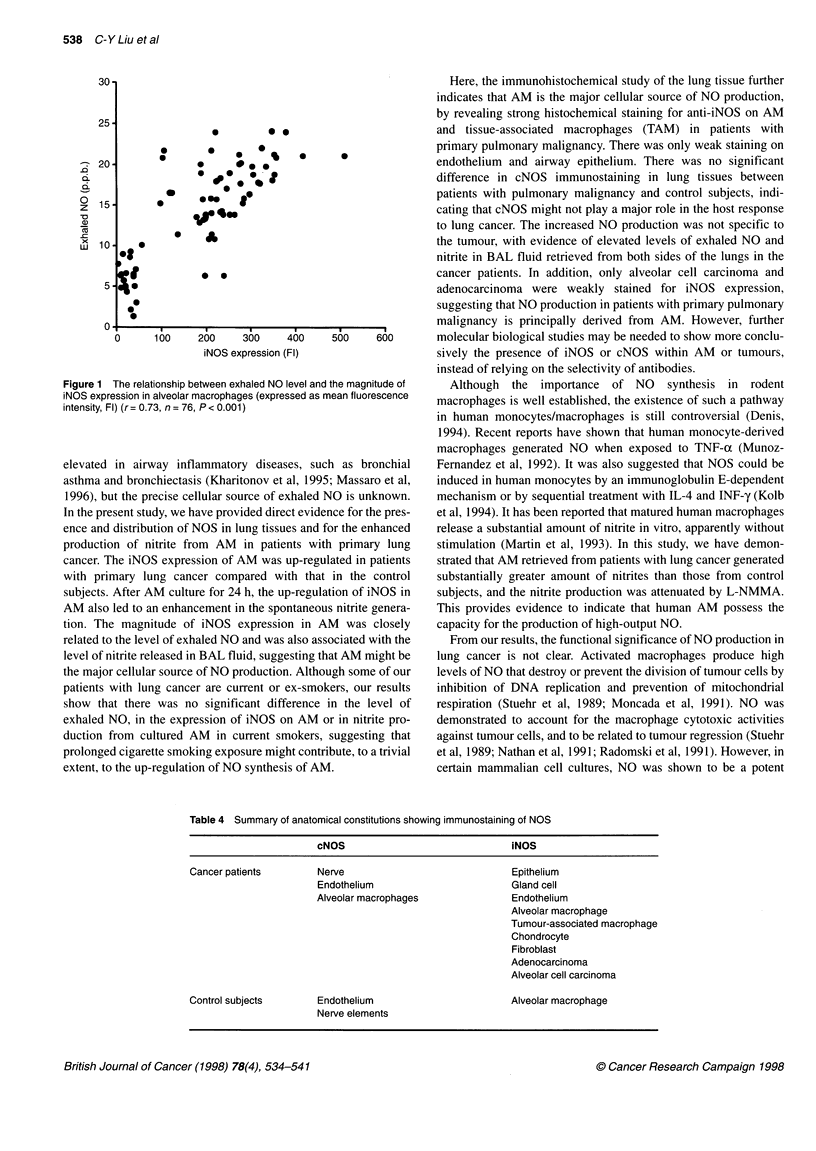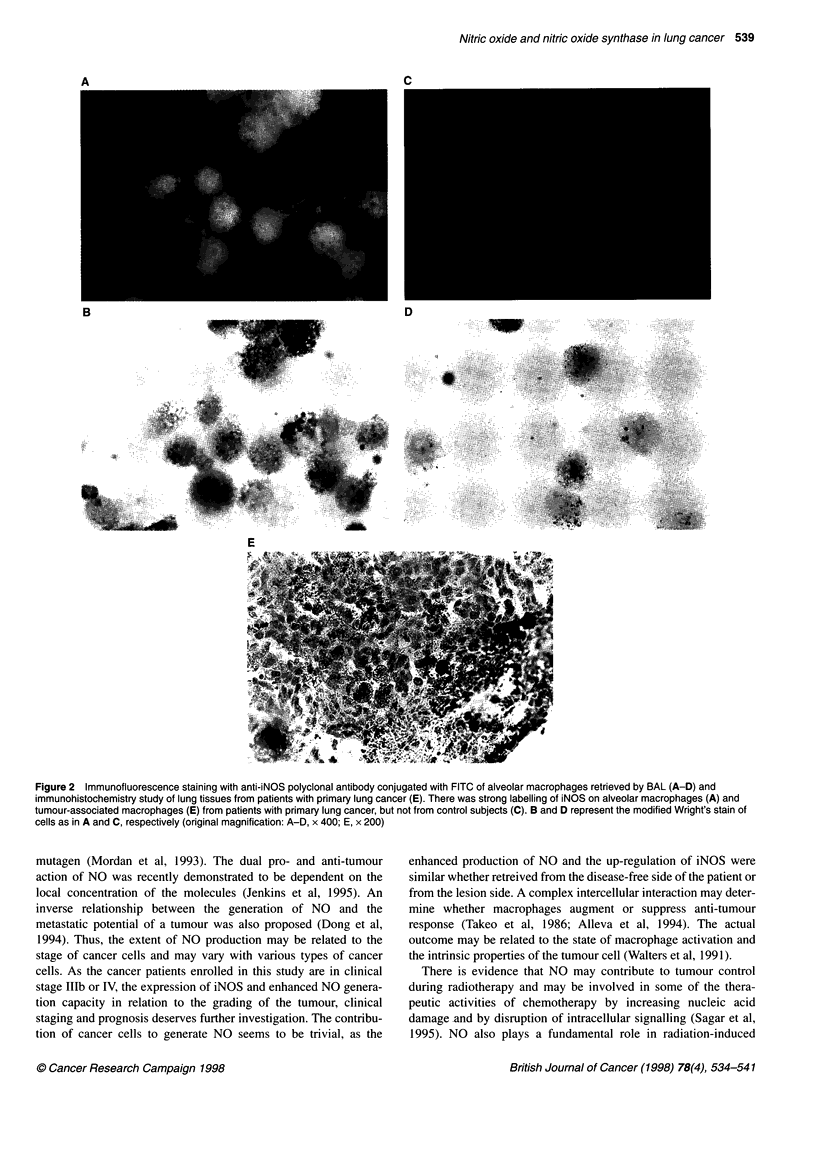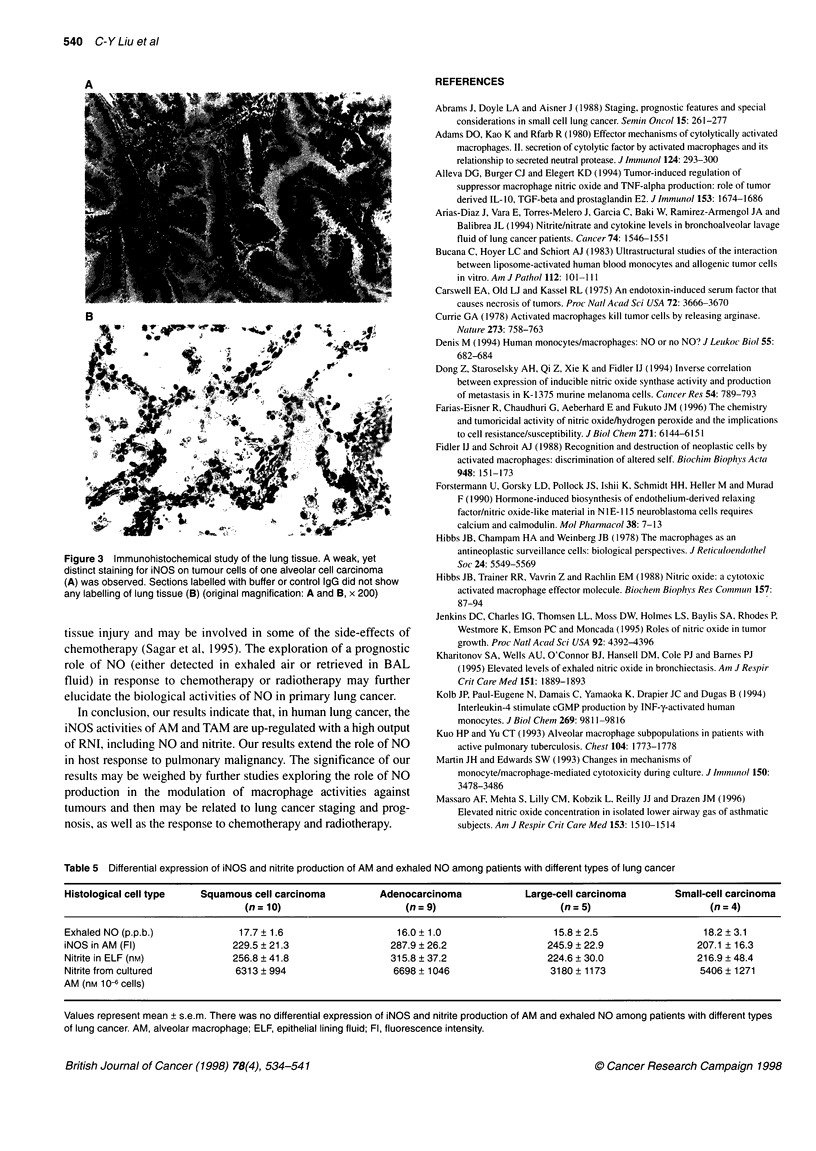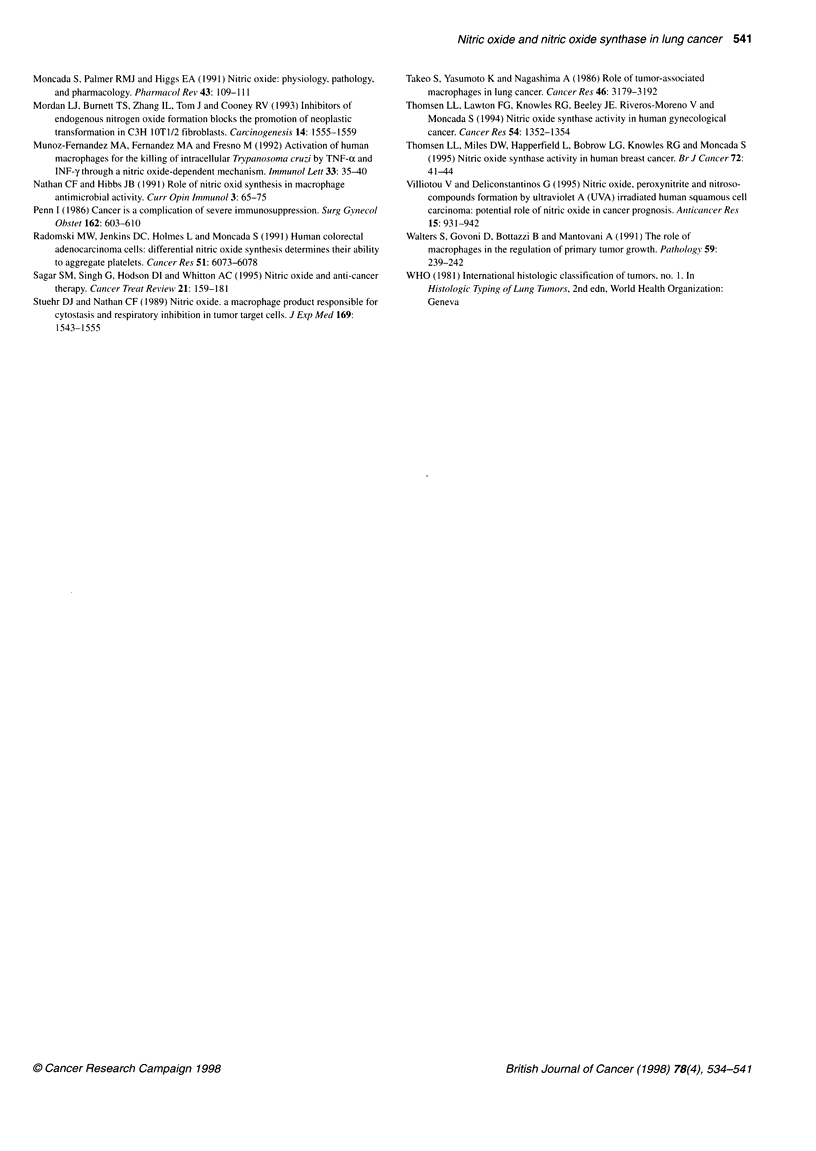# Increased level of exhaled nitric oxide and up-regulation of inducible nitric oxide synthase in patients with primary lung cancer.

**DOI:** 10.1038/bjc.1998.528

**Published:** 1998-08

**Authors:** C. Y. Liu, C. H. Wang, T. C. Chen, H. C. Lin, C. T. Yu, H. P. Kuo

**Affiliations:** Department of Thoracic Medicine II, Chang Gung Memorial Hospital, Taipei, Taiwan.

## Abstract

**Images:**


British Journal of Cancer (1998) 78(4), 534–541
© 1998 Cancer Research Campaign

### Increased level of exhaled nitric oxide and up-regulation of inducible nitric oxide synthase in patients with primary lung cancer

C-Y Liu[1], C-H Wang[1], T-C Chen[2], H-C Lin[1], C-T Yu[1] and H-P Kuo[1]

Departments of [1]Thoracic Medicine II and [2]Pathology, Chang Gung Memorial Hospital, Taipei, Taiwan

**Summary** Monocyte–macrophage series have an important role in host surveillance against cancer. The cytotoxic/cytostatic activity of macrophages is, to a great extent, attributed to the up-regulation of inducible nitric oxide synthase (iNOS) and production of nitric oxide (NO). Here, in 28 patients with primary lung cancer and 20 control subjects, we measured the concentration of exhaled NO and nitrite in epithelial lining fluid (ELF) using a chemiluminescence NO analyser, and studied NOS expression in alveolar macrophages (AM) and lung tissues by flow cytometry; immunohistochemical analysis was also undertaken. The mean fluorescence intensity (FI) of iNOS expression in AM was significantly increased in patients with lung cancer (tumour side 263.5 ± 15.2 FI, normal side 232.4 ± 18.6 FI; $n = 28$) compared with that in control subjects (27.3 ± 3.2 FI; $n = 20$, $P < 0.001$). The level of exhaled NO from cancer patients (16.9 ± 0.9 p.p.b.; $n = 28$) was significantly higher than that in the control group (6.0 ± 0.5 p.p.b.; $n = 20$, $P < 0.001$). The level of nitrite was also significantly higher in ELF from cancer patients (tumour side 271.1 ± 28.9 nM and normal side 257.4 ± 19.6 nM vs control subjects 32.9 ± 4.1 nM; $P < 0.001$). The intensity of iNOS expression in AM was correlated with the level of exhaled NO ($r_s = 0.73$, $n = 76$, $P < 0.001$) and the nitrite released in ELF ($r_s = 0.56$, $n = 76$, $P < 0.001$). The nitrite generation of cultured AM from patients with lung cancer was significantly enhanced compared with that of control subjects after culture for 24 h (tumour side 5.75 ± 0.69 and normal side 5.68 ± 0.58 μM per $10^6$ cells vs control group 38.3 ± 3.6 nM per $10^6$ cells; $P < 0.001$). The distribution of iNOS was identified in AM, tumour-associated macrophages, endothelium, chondrocytes, airway epithelium of both lungs and malignant cells (adenocarcinoma and alveolar cell carcinoma) of cancer patients. cNOS was labelled in alveolar macrophages, endothelial cells and nerve elements from lung tissue. Our results indicate that, in patients with primary lung cancer, the production of NO from alveolar macrophages was increased as a result of the up-regulation of iNOS activity. The increased NO production was not specific to the tumour side and might be attributed to the tumour-associated non-specific immunological and inflammatory processes of the host.

**Keywords:** lung cancer; alveolar macrophage; nitric oxide; nitric oxide synthase; nitrite; cytotoxicity

The host defence mechanism is important in the development and growth of tumours. The incidence of malignancy is reported to be increased in subjects with compromised immunity (Penn, 1986). The complex defence and immunological mechanisms against cancer contain several types of cells, including macrophages (Fidler et al, 1988) and mediators, such as nitric oxide (NO) (Farias-Eisner et al, 1996).

Macrophages have a role in host surveillance against cancer (Hibbs et al, 1978). Macrophages can be activated both in vivo and in vitro to kill tumour cells. The oncolytic activity of macrophages is either mediated by direct macrophage-to-tumour cell contact (Bucana et al, 1983) or attributed to the production of soluble tumour cytotoxic factors, such as tumour necrosis factor-α (TNF-α), interleukin I (IL-1), IL-6, cytolytic proteases, arginases, lysosomal enzymes, prostaglandins, oxygen radicals and reactive nitrogen species, particularly NO (Carswell et al, 1975; Currie, 1978; Adams et al, 1980; Hibbs et al, 1988; Nathan, 1991). The cytotoxicity of activated macrophages against tumour target cells is dependent on the synthesis of NO (Hibbs et al, 1988). The production of NO from activated macrophages destroys or prevents tumour cell division by inhibition of DNA replication and restraint of mitochondrial respiration (Stuehr et al, 1989; Moncada et al, 1991). Furthermore, the constitutive and inducible NOS is also present in several types of tumour cells, including colorectal adenocarcinoma (Radomski et al, 1991), gynecological carcinoma (Thomsen et al, 1994, 1995), neuroblastoma (Forstermann et al, 1990) and dermal squamous cell carcinoma (Villiotou et al, 1995). Thus, NO production is enhanced in patients with malignancy either directly from tumour cells or from activated macrophages as host defence mechanisms against tumour cells.

Recently, it has also been reported that tumour-associated NO production increases in lung cancer, as suggested indirectly by an increased level of nitrite/nitrate in the bronchoalveolar lavage (BAL) fluid of lung cancer patients (squamous cell carcinoma) (Arias-Diaz et al, 1994). However, the cellular source of NO generation in lung cancer is still unknown. Therefore, we felt it necessary to further characterize the role of NO in the biology of lung cancer. In this study, we have measured NO levels in exhaled air and explored NOS localization within lung tissues as well as NO production capacity from alveolar macrophages. The distribution of NOS expression in lung tissue was also compared in different types of lung cancers.

*Received 2 May 1997*
*Revised 15 December 1997*
*Accepted 30 December 1997*

*Correspondence to:* H-P Kuo, Department of Thoracic Medicine II, Chang Gung Memorial Hospital, 199 Tun-Hwa N. Rd, Taipei, Taiwan

### MATERIALS AND METHODS

### Patients

Twenty-eight patients with bronchogenic carcinoma proven histologically before treatment, without other systemic diseases, were enrolled in this study. To avoid the possible confounding effects of cigarette smoking on exhaled NO levels or NOS activity, 20 of them were non-smokers selected intentionally from our chest clinics. Of the control subjects, five were healthy volunteers and eight had received bronchoscopy because of haemoptysis. Another five were found to have single nodular lesions on chest radiograms, and the remaining two presented with coughs and local wheezing on physical examination. All of them had no abnormal findings at bronchoscopic, microbiological and cytological investigation. Eight current smokers with lung cancer and nine of the controls were also recruited into this study to compare the difference between smokers and non-smokers. All subjects had had no upper respiratory tract infection within the last 6 weeks. None of them were taking antibiotics, immunosuppressants or other regular medication at the time of evaluation. Informed consents were obtained from all subjects.

All patients with malignancy received complete staging, including computerized tomography of chest, liver echography and bone scan. The International TNM staging system was applied for staging the patients with non-small-cell lung cancers and a simple two-stage system for those with small-cell lung cancer, which classified patients as having limited disease (LD) or extensive disease (ED) (Abrams et al, 1988).

### Histology and nomenclature of lung cancer

Tumour types were determined by histological assessment of haematoxylin- and eosin-stained tissue sections. The carcinomas were classified into squamous carcinoma, small-cell carcinoma, adenocarcinoma (including alveolar cell carcinoma), undifferentiated large-cell carcinoma and unclassified carcinoma according to the WHO classification (WHO, 1981).

### Measurement of exhaled nitric oxide

Exhaled NO was measured using a chemiluminescence analyser (NOA Model 280; Sievers, Boulder, CO, USA), which was adapted for on-line recording of NO concentration. To decrease the confounding effect of environment, for all subjects, exhaled NO was sampled after inspiring NO-free gas (21% $O_2$ and 79% $N_2$) for 3 min. Then all subjects were asked to perform a slow vital-capacity manoeuvre over 30–45 s into wide-bore Teflon tubing, and NO was sampled continuously at a flow rate of 200 ml $min^{-1}$. Subjects wore nose clips and were in sitting or standing positions during the measurement of the exhaled NO level. A high-flow mask with a one-way valve was worn when measuring the endotracheal level of NO at the orifices of bilateral main bronchi by bronchoscopy. Results were displayed on a chart recorder. Three successive peak values were recorded and the mean values analysed.

### Preparation of lung cells

Bronchoalveolar lavage (BAL) was performed using three aliquots (50 ml each) of 0.9% saline solution as described previously (Kuo et al, 1993). The total cell and differential cell counts were determined. The BAL fluid was centrifuged at 600 ***g*** for 20 min at 4°C, and the cell pellet was washed sequentially and resuspended in RPMI-1640 (Gibco, Grand Island, NY, USA) supplemented with 5% heat-inactivated fetal calf serum (FCS, Flow Laboratories, Paisley, UK) at $10^6$ cells $ml^{-1}$. The cell viability was determined by trypan blue exclusion.

### Culture of alveolar macrophages

Alveolar macrophages were placed in 75-$cm^2$ plastic culture dishes in RPMI-1640, allowed to adhere for 90 min and then washed three times with warmed RPMI to remove non-adherent cells. Adherent cells were scraped off with a sterile rubber policeman and then resuspended ($10^6$ cells $ml^{-1}$) in RPMI-1640 containing 5% FCS, 100 U $ml^{-1}$ penicillin and 100 µg $ml^{-1}$ streptomycin. Then the AM were placed in 12-well Petri dishes at $10^6$ cells $ml^{-1}$ for 24 h at 37°C, 5% carbon dioxide. The culture supernatant was collected and frozen at –72°C before measuring RNI production. To determine that the generation of RNI was specific for NO production, alveolar macrophages were cultured in the presence or absence of a NOS inhibitor ($N^G$-monomethyl-L-arginine, L-NMMA, 1 mM) (Calbiochem, La Jolla, CA, USA). The supernatant of culture medium was harvested and stored at –72°C before analysis.

### Measurement of iNOS expression in alveolar macrophages

Lavaged cells at 1 × $10^6$ cells $ml^{-1}$ were fixed with 100 µl of 4% paraformaldehyde at room temperature for 10 min and then washed twice with phosphate-buffered saline (PBS) pH 7.0. The cell pellet was resuspended in 20 µl of 3.7% n-octyl-β-D-glucopyranoside (OG, Sigma) and then incubated for 5 min at room temperature before being washed twice with PBS. Thereafter cells were incubated with anti-iNOS rabbit polyclonal (Transduction Laboratories, Lexington, KY) diluted 1:25 in PBS (20 µl) in the dark for 1 h at 4°C. After being washed twice with PBS, the cell pellet was labelled with 5 µl of fluorescein isothiocyanate (FITC) conjugated swine anti-rabbit IgG (Dakopatts, Glostrup, Denmark) and placed in the dark for 30 min at 4°C, and subsequently analysed by flow cytometry after two extensive washings with cold PBS containing 5% FCS. Analysis of the fluorescence intensity of iNOS in alveolar macrophages was performed with a FACScan flow cytometer (Becton Dickson, Mountain View, CA, USA) and Cell-Quest software (Becton Dickson). Controls were used to give a measure of non-specific binding using FITC-conjugated $F(ab)_2$ fragments of rabbit anti-mouse immunoglobulin (Dako, Kyoto, Japan). Results were expressed as a mean fluorescence intensity in arbitrary units transformed to a linear scale from the $log_{10}$ channel number of mean fluorescence, for a particular cell marker.

### Measurement of nitrite in BAL fluid and supernatant of AM culture

To measure the concentration of nitrite, 50 µl of lavaged fluid or culture media supernatant were added to the purge vessel containing 5 ml of a reducing solution (1% potassium iodide in acetic acid) that converts nitrite to nitric oxide. Quantification of the NO formed from reactive nitrogen intermediate (RNI) was

© Cancer Research Campaign 1998

determined from the specific chemiluminescence resulting from the reduction of NO with ozone using the chemiluminescence analyser (Sievers NOA Model 280, Boulder, CO, USA). Conversion of standard mixtures of nitrite and nitrate solutions to NO was 94% compared with calibrated standards of NO gas.

### NOS localization by immunohistochemical study

Sections (4 μm) were cut from formalin-fixed, paraffin-embedded lung tissues. Two antibodies were used for NOS detection: (1) a polyclonal rabbit antibody to iNOS from murine macrophages that immunoreacted with human iNOS (Anti-macNOS; Transduction Laboratories, Lexington, KY, USA) and (2) two monoclonal mouse antibodies to cNOS from human endothelium and brain tissue (Anti-ENOS and Anti-BNOS; Transduction Laboratories, Lexington, KY, USA). Non-specific mouse IgG was used as a control. After being de-waxed in xylene and rinsed in absolute alcohol, sections were incubated in 3% hydrogen peroxide in methanol for 30 min to quench endogenous peroxidase. Then the sections were microwaved in citric acid buffer with 0.1% (v/v) Triton X 100 for 5 min to enhance the antigen exposure and incubated in 0.2% (v/v) normal swine serum (Dako, CA, USA) for 30 min to block the positive and negative charges of tissues. Afterwards, the sections were subjected to 1-h incubation with primary specific anti-NOS antibody (diluted 1:200) or non-specific purified mouse IgG (diluted 1:200) as a control. Antibody labelling was subsequently visualized using an avidin–biotin complex method (LSAB 2 kit, Dako; DAB peroxidase substrate kit, Vector Laboratories, Burlingame, CA, USA). The slides were then counterstained with haematoxylin, dehydrated through graded alcohol and xylene, mounted and coverslipped.

### Statistical analysis

Standard formulas were used for the analysis. Data did not approximate a Gaussian distribution, whereby the mean value did not approximate the median value. Non-parametric statistical analyses were therefore used, and the probability of differences between groups was initially assessed using Kruskal–Wallis analysis. The number of patients in some groups was too small to allow for a strict median test between groups, and subsequent analysis was performed using the Mann–Whitney *U*-test (two-tailed) to assess the significance of differences between groups. To minimize the possibility of obtaining chance significance as a result of multiple comparisons, preplanned comparisons between specific groups were made and the significant values confirmed using Newman–Keuls analysis. Relationships between mean fluorescence intensity of iNOS expression in AM, the levels of exhaled NO and nitrite in BAL fluid and supernatant of AM culture were investigated using Spearman's rank test. Data represent means ± s.e.m. The null hypothesis was rejected at $P < 0.05$.

### RESULTS

### Patients

Twenty-eight patients with primary lung cancer (18 male and 10 female, 34–84 years of age) and 20 control subjects (13 male and 7 female, 21–63 years of age) were enrolled in this study. The pathology and clinical characteristics of these patients are shown in Table 1.

**Table 1** Pathology and clinical characteristics of patients with primary lung cancer

| Patient characteristics | Patient no. ($n = 28$) |
|---|---|
| Histological cell type | |
| Non-small-cell carcinoma | 24 |
| Squamous cell carcinoma | 10 |
| Adenocarcinoma | 9 |
| Large-cell carcinoma | 5 |
| Small-cell carcinoma | 4 |
| Clinical staging | |
| Non-small-cell carcinoma | |
| Stage IIIb | 9 |
| Stage IV | 15 |
| Small-cell carcinoma | |
| Limited stage | 1 |
| Extensive stage | 3 |
| Performance status | |
| ECOG scale 1 | 15 |
| ECOG scale 2 | 10 |
| ECOG scale 3 | 3 |

### Cellular profiles in BAL

The total cell counts retrieved in BAL fluid in the cancer patients, either from the tumour side or from the normal side, were significantly higher than those in the control group. The recovery rate was significantly lower in the tumour side of cancer patients, and viability was significantly higher in the control group. There was no significant difference in the differential cell counts between the two groups (Table 2).

### The level of exhaled NO and expression of iNOS in alveolar macrophages

The level of exhaled NO from the cancer patients was 16.9 ± 0.9 p.p.b. ($n = 28$), which was significantly higher than that from the control subjects (6.0 ± 0.5 p.p.b., $n = 20$, $P < 0.001$) (Table 3). There was no significant difference in NO between smokers and non-smokers in either group (Table 3). The difference between the cancer patients and the control group was still significant regardless of smoking exposure. NO measured by bronchoscopy in the cancer patients showed no significant difference between the normal side and the tumour side (Table 3). The magnitude of iNOS expression in AM was significantly increased in those patients with pulmonary malignancy, retrieved either from the tumour side or from the normal side, compared with that in the control subjects (Table 3). The level of exhaled NO was correlated with the intensity of iNOS expression in AM ($r_s = 0.73$, $n = 76$, $P < 0.001$) (Figure 1).

### Nitrite released in BAL fluid and supernatant of AM culture

The level of nitrite was significantly higher in BAL fluid from the cancer patients, either from the tumour side (271.1 ± 28.9 nM) or from the normal side (257.4 ± 19.6 nM) ($n = 28$), than in that from the control group (32.9 ± 4.1 nM, $n = 20$, $P < 0.001$) (Table 3). The intensity of iNOS expression in AM was significantly correlated with the nitrite level retrieved in BAL fluid ($r_s = 0.56$, $n = 76$, $P < 0.001$), as well as with the nitrite generation after a 24-h

 © Cancer Research Campaign 1998

**Table 2** Total cell counts and cellular populations recovered by bronchoalveolar lavage in patients with primary lung cancer and control subjects

| | **Patients ($n$ = 28)** | | **Control subjects ($n$ = 20)** |
|---|---|---|---|
| | **Tumour side** | **Normal side** | |
| Total cell count (× $10^6$) | 8.28 ± 0.73[a] | 8.72 ± 0.90[a] | 4.12 ± 0.41 |
| Recovered rate (% v/v) | 56.0 ± 1.9[b] | 65.4 ± 2.1 | 67.4 ± 1.9 |
| Cell viability (%) | 88.9 ± 1.1[a] | 91.7 ± 1.0[a] | 95.1 ± 0.47 |
| Differential count | | | |
| Alveolar macrophages (%) | 85.9 ± 2.3 | 84.5 ± 1.9 | 89.0 ± 1.0 |
| Lymphocytes (%) | 10.3 ± 1.8 | 12.2 ± 2.3 | 9.9 ± 1.0 |
| Neutrophils (%) | 3.8 ± 1.3 | 3.3 ± 1.1 | 1.1 ± 0.2 |

Values represent mean ± s.e.m. [a]$P < 0.05$ compared with control group. [b]$P < 0.05$ compared with normal side of cancer patient and control group.

**Table 3** NOS activity in both lungs of patients with lung cancer ($n$ = 28), as measured by exhaled NO level, nitrite concentration in ELF and supernatant of AM culture, fluorescence intensity of iNOS in AM and LNMMA inhibition study, compared with that in control subjects ($n$ = 20)

| | **Patients (smokers, $n$ = 8)** | | **Control subjects (smokers, $n$ = 9)** |
|---|---|---|---|
| | **Tumour side** | **Normal side** | |
| NO (p.p.b.) | | | |
| Exhaled level | 16.9 ± 0.9[a] | | 6.0 ± 0.5 |
| | (17.5 ± 2.1[a]) | | (6.5 ± 0.6) |
| Endobronchial level | 16.8 ± 0.8[a] | 16.2 ± 0.6[a] | |
| | (17.3 ± 1.1[a]) | (16.5 ± 1.2[a]) | |
| iNOS in AM (FI) | 263.5 ± 15.2[a] | 232.4 ± 18.6[a] | 27.3 ± 3.2 |
| | (261.1 ± 18.8[a]) | (250.2 ± 21.4[a]) | (29.0 ± 4.9) |
| Nitrite in ELF (nM) | 271.1 ± 28.9[a] | 257.4 ± 19.6[a] | 32.9 ± 4.1 |
| | (268.7 ± 21.7[a]) | (251.5 ± 23.2[a]) | (37.5 ± 5.5) |
| Nitrite from cultured AM (nM $10^{-6}$ cells) | 5750 ± 690[a] | 5677 ± 579[a] | 38.3 ± 3.6 |
| | (6403 ± 1381[a]) | (5601 ± 1002) | (43.0 ± 6.1) |
| Nitrite from cultured AM +LNMMA (nM $10^{-6}$ cells) | 3462 ± 512[a,b] | 3612 ± 471[a,b] | 32.4 ± 2.6 |
| | ($n$ = 23) | ($n$ = 23) | ($n$ = 18) |
| | (3596 ± 742[a,b]) | (2274 ± 396[a,b]) | (32.6 ± 2.9) |

Values represent mean ± s.e.m. [a]$P < 0.001$ compared with control subjects. [b]$P < 0.05$ compared with corresponding group without LNMMA treatment. AM, alveolar macrophage; ELF, epithelial lining fluid; FI, fluorescence intensity. Data in parentheses are from smokers in either group.

culture of AM ($r_s = 0.49$, $n = 76$, $P < 0.001$). The nitrite generation by cultured AM from the patients with lung cancer was significantly enhanced compared with that from the control subjects (Table 3). The spontaneous production of nitrite from cultured AM in the cancer patients was significantly inhibited by L-NMMA, at 1 mmol ml$^{-1}$ ($n = 46$, $P < 0.05$). In contrast, there was no significant change in the production of nitrite from cultured AM of the control subjects (Table 3). There was no significant difference between smokers and non-smokers in either group (Table 3).

### Immunofluorescence and immunohistochemical staining of NOS in the lung

Inducible and constitutive forms of NOS were identified in many constitutive elements and inflammatory cells within the lung cancer tissues (Table 4, Figures 2 and 3). AM and tissue-associated macrophages from patients with primary lung cancer, but not from control subjects, were strongly labelled with anti-iNOS polyclonal antibody (Figure 2A–E). There was weak, yet distinct staining for iNOS on tumour cells of two adenocarcinomas and one alveolar cell carcinoma (Figure 3A). Immunolabelling with anti-iNOS and cNOS could not detect antigens on tumour cells of squamous carcinomas, large-cell carcinomas, small-cell carcinomas and five other adenocarcinomas. Sections labelled with buffer or control IgG did not show any labelling of lung tissue (Figure 3B).

### Differential expression of iNOS, nitrite production and exhaled NO among patients with different types of lung cancer

There was no significant difference in the exhaled NO concentration, fluorescence intensity of iNOS activity in AM, nitrite level in BAL fluid and supernatant of AM culture between patients with different types of lung cancer (Table 5).

### DISCUSSION

In this study, we have demonstrated that the level of exhaled NO was greater from patients with primary lung cancer than that from control subjects. Exhaled NO has been demonstrated to be

*© Cancer Research Campaign 1998*

**Figure 1** The relationship between exhaled NO level and the magnitude of iNOS expression in alveolar macrophages (expressed as mean fluorescence intensity, FI) ($r = 0.73$, $n = 76$, $P < 0.001$)

elevated in airway inflammatory diseases, such as bronchial asthma and bronchiectasis (Kharitonov et al, 1995; Massaro et al, 1996), but the precise cellular source of exhaled NO is unknown. In the present study, we have provided direct evidence for the presence and distribution of NOS in lung tissues and for the enhanced production of nitrite from AM in patients with primary lung cancer. The iNOS expression of AM was up-regulated in patients with primary lung cancer compared with that in the control subjects. After AM culture for 24 h, the up-regulation of iNOS in AM also led to an enhancement in the spontaneous nitrite generation. The magnitude of iNOS expression in AM was closely related to the level of exhaled NO and was also associated with the level of nitrite released in BAL fluid, suggesting that AM might be the major cellular source of NO production. Although some of our patients with lung cancer are current or ex-smokers, our results show that there was no significant difference in the level of exhaled NO, in the expression of iNOS on AM or in nitrite production from cultured AM in current smokers, suggesting that prolonged cigarette smoking exposure might contribute, to a trivial extent, to the up-regulation of NO synthesis of AM.

Here, the immunohistochemical study of the lung tissue further indicates that AM is the major cellular source of NO production, by revealing strong histochemical staining for anti-iNOS on AM and tissue-associated macrophages (TAM) in patients with primary pulmonary malignancy. There was only weak staining on endothelium and airway epithelium. There was no significant difference in cNOS immunostaining in lung tissues between patients with pulmonary malignancy and control subjects, indicating that cNOS might not play a major role in the host response to lung cancer. The increased NO production was not specific to the tumour, with evidence of elevated levels of exhaled NO and nitrite in BAL fluid retrieved from both sides of the lungs in the cancer patients. In addition, only alveolar cell carcinoma and adenocarcinoma were weakly stained for iNOS expression, suggesting that NO production in patients with primary pulmonary malignancy is principally derived from AM. However, further molecular biological studies may be needed to show more conclusively the presence of iNOS or cNOS within AM or tumours, instead of relying on the selectivity of antibodies.

Although the importance of NO synthesis in rodent macrophages is well established, the existence of such a pathway in human monocytes/macrophages is still controversial (Denis, 1994). Recent reports have shown that human monocyte-derived macrophages generated NO when exposed to TNF-α (Munoz-Fernandez et al, 1992). It was also suggested that NOS could be induced in human monocytes by an immunoglobulin E-dependent mechanism or by sequential treatment with IL-4 and INF-γ (Kolb et al, 1994). It has been reported that matured human macrophages release a substantial amount of nitrite in vitro, apparently without stimulation (Martin et al, 1993). In this study, we have demonstrated that AM retrieved from patients with lung cancer generated substantially greater amount of nitrites than those from control subjects, and the nitrite production was attenuated by L-NMMA. This provides evidence to indicate that human AM possess the capacity for the production of high-output NO.

From our results, the functional significance of NO production in lung cancer is not clear. Activated macrophages produce high levels of NO that destroy or prevent the division of tumour cells by inhibition of DNA replication and prevention of mitochondrial respiration (Stuehr et al, 1989; Moncada et al, 1991). NO was demonstrated to account for the macrophage cytotoxic activities against tumour cells, and to be related to tumour regression (Stuehr et al, 1989; Nathan et al, 1991; Radomski et al, 1991). However, in certain mammalian cell cultures, NO was shown to be a potent

**Table 4** Summary of anatomical constitutions showing immunostaining of NOS

| | cNOS | iNOS |
|---|---|---|
| Cancer patients | Nerve<br>Endothelium<br>Alveolar macrophages | Epithelium<br>Gland cell<br>Endothelium<br>Alveolar macrophage<br>Tumour-associated macrophage<br>Chondrocyte<br>Fibroblast<br>Adenocarcinoma<br>Alveolar cell carcinoma |
| Control subjects | Endothelium<br>Nerve elements | Alveolar macrophage |

 © Cancer Research Campaign 1998

**Figure 2** Immunofluorescence staining with anti-iNOS polyclonal antibody conjugated with FITC of alveolar macrophages retrieved by BAL (**A–D**) and immunohistochemistry study of lung tissues from patients with primary lung cancer (**E**). There was strong labelling of iNOS on alveolar macrophages (**A**) and tumour-associated macrophages (**E**) from patients with primary lung cancer, but not from control subjects (**C**). **B** and **D** represent the modified Wright's stain of cells as in **A** and **C**, respectively (original magnification: A–D, × 400; E, × 200)

mutagen (Mordan et al, 1993). The dual pro- and anti-tumour action of NO was recently demonstrated to be dependent on the local concentration of the molecules (Jenkins et al, 1995). An inverse relationship between the generation of NO and the metastatic potential of a tumour was also proposed (Dong et al, 1994). Thus, the extent of NO production may be related to the stage of cancer cells and may vary with various types of cancer cells. As the cancer patients enrolled in this study are in clinical stage IIIb or IV, the expression of iNOS and enhanced NO generation capacity in relation to the grading of the tumour, clinical staging and prognosis deserves further investigation. The contribution of cancer cells to generate NO seems to be trivial, as the enhanced production of NO and the up-regulation of iNOS were similar whether retreived from the disease-free side of the patient or from the lesion side. A complex intercellular interaction may determine whether macrophages augment or suppress anti-tumour response (Takeo et al, 1986; Alleva et al, 1994). The actual outcome may be related to the state of macrophage activation and the intrinsic properties of the tumour cell (Walters et al, 1991).

There is evidence that NO may contribute to tumour control during radiotherapy and may be involved in some of the therapeutic activities of chemotherapy by increasing nucleic acid damage and by disruption of intracellular signalling (Sagar et al, 1995). NO also plays a fundamental role in radiation-induced

© Cancer Research Campaign 1998

A

Figure 3 Immunohistochemical study of the lung tissue. A weak, yet distinct staining for iNOS on tumour cells of one alveolar cell carcinoma (A) was observed. Sections labelled with buffer or control IgG did not show any labelling of lung tissue (B) (original magnification: A and B, × 200)

tissue injury and may be involved in some of the side-effects of chemotherapy (Sagar et al, 1995). The exploration of a prognostic role of NO (either detected in exhaled air or retrieved in BAL fluid) in response to chemotherapy or radiotherapy may further elucidate the biological activities of NO in primary lung cancer.

In conclusion, our results indicate that, in human lung cancer, the iNOS activities of AM and TAM are up-regulated with a high output of RNI, including NO and nitrite. Our results extend the role of NO in host response to pulmonary malignancy. The significance of our results may be weighed by further studies exploring the role of NO production in the modulation of macrophage activities against tumours and then may be related to lung cancer staging and prognosis, as well as the response to chemotherapy and radiotherapy.

### REFERENCES

Abrams J, Doyle LA and Aisner J (1988) Staging, prognostic features and special considerations in small cell lung cancer. *Semin Oncol* **15**: 261–277

Adams DO, Kao K and Rfarb R (1980) Effector mechanisms of cytolytically activated macrophages. II. secretion of cytolytic factor by activated macrophages and its relationship to secreted neutral protease. *J Immunol* **124**: 293–300

Alleva DG, Burger CJ and Elegert KD (1994) Tumor-induced regulation of suppressor macrophage nitric oxide and TNF-alpha production: role of tumor derived IL-10, TGF-beta and prostaglandin E2. *J Immunol* **153**: 1674–1686

Arias-Diaz J, Vara E, Torres-Melero J, Garcia C, Baki W, Ramirez-Armengol JA and Balibrea JL (1994) Nitrite/nitrate and cytokine levels in bronchoalveolar lavage fluid of lung cancer patients. *Cancer* **74**: 1546–1551

Bucana C, Hoyer LC and Schiort AJ (1983) Ultrastructural studies of the interaction between liposome-activated human blood monocytes and allogenic tumor cells in vitro. *Am J Pathol* **112**: 101–111

Carswell EA, Old LJ and Kassel RL (1975) An endotoxin-induced serum factor that causes necrosis of tumors. *Proc Natl Acad Sci USA* **72**: 3666–3670

Currie GA (1978) Activated macrophages kill tumor cells by releasing arginase. *Nature* **273**: 758–763

Denis M (1994) Human monocytes/macrophages: NO or no NO? *J Leukoc Biol* **55**: 682–684

Dong Z, Staroselsky AH, Qi Z, Xie K and Fidler IJ (1994) Inverse correlation between expression of inducible nitric oxide synthase activity and production of metastasis in K-1375 murine melanoma cells. *Cancer Res* **54**: 789–793

Farias-Eisner R, Chaudhuri G, Aeberhard E and Fukuto JM (1996) The chemistry and tumoricidal activity of nitric oxide/hydrogen peroxide and the implications to cell resistance/susceptibility. *J Biol Chem* **271**: 6144–6151

Fidler IJ and Schroit AJ (1988) Recognition and destruction of neoplastic cells by activated macrophages: discrimination of altered self. *Biochim Biophys Acta* **948**: 151–173

Forstermann U, Gorsky LD, Pollock JS, Ishii K, Schmidt HH, Heller M and Murad F (1990) Hormone-induced biosynthesis of endothelium-derived relaxing factor/nitric oxide-like material in N1E-115 neuroblastoma cells requires calcium and calmodulin. *Mol Pharmacol* **38**: 7–13

Hibbs JB, Champam HA and Weinberg JB (1978) The macrophages as an antineoplastic surveillance cells: biological perspectives. *J Reticuloendothel Soc* **24**: 5549–5569

Hibbs JB, Trainer RR, Vavrin Z and Rachlin EM (1988) Nitric oxide: a cytotoxic activated macrophage effector molecule. *Biochem Biophys Res Commun* **157**: 87–94

Jenkins DC, Charles IG, Thomsen LL, Moss DW, Holmes LS, Baylis SA, Rhodes P, Westmore K, Emson PC and Moncada (1995) Roles of nitric oxide in tumor growth. *Proc Natl Acad Sci USA* **92**: 4392–4396

Kharitonov SA, Wells AU, O'Connor BJ, Hansell DM, Cole PJ and Barnes PJ (1995) Elevated levels of exhaled nitric oxide in bronchiectasis. *Am J Respir Crit Care Med* **151**: 1889–1893

Kolb JP, Paul-Eugene N, Damais C, Yamaoka K, Drapier JC and Dugas B (1994) Interleukin-4 stimulate cGMP production by INF-γ-activated human monocytes. *J Biol Chem* **269**: 9811–9816

Kuo HP and Yu CT (1993) Alveolar macrophage subpopulations in patients with active pulmonary tuberculosis. *Chest* **104**: 1773–1778

Martin JH and Edwards SW (1993) Changes in mechanisms of monocyte/macrophage-mediated cytotoxicity during culture. *J Immunol* **150**: 3478–3486

Massaro AF, Mehta S, Lilly CM, Kobzik L, Reilly JJ and Drazen JM (1996) Elevated nitric oxide concentration in isolated lower airway gas of asthmatic subjects. *Am J Respir Crit Care Med* **153**: 1510–1514

**Table 5** Differential expression of iNOS and nitrite production of AM and exhaled NO among patients with different types of lung cancer

| Histological cell type | Squamous cell carcinoma (*n* = 10) | Adenocarcinoma (*n* = 9) | Large-cell carcinoma (*n* = 5) | Small-cell carcinoma (*n* = 4) |
|---|---|---|---|---|
| Exhaled NO (p.p.b.) | 17.7 ± 1.6 | 16.0 ± 1.0 | 15.8 ± 2.5 | 18.2 ± 3.1 |
| iNOS in AM (FI) | 229.5 ± 21.3 | 287.9 ± 26.2 | 245.9 ± 22.9 | 207.1 ± 16.3 |
| Nitrite in ELF (nM) | 256.8 ± 41.8 | 315.8 ± 37.2 | 224.6 ± 30.0 | 216.9 ± 48.4 |
| Nitrite from cultured AM (nM $10^{-6}$ cells) | 6313 ± 994 | 6698 ± 1046 | 3180 ± 1173 | 5406 ± 1271 |

Values represent mean ± s.e.m. There was no differential expression of iNOS and nitrite production of AM and exhaled NO among patients with different types of lung cancer. AM, alveolar macrophage; ELF, epithelial lining fluid; FI, fluorescence intensity.

© Cancer Research Campaign 1998

Moncada S, Palmer RMJ and Higgs EA (1991) Nitric oxide: physiology, pathology, and pharmacology. *Pharmacol Rev* **43**: 109–111

Mordan LJ, Burnett TS, Zhang IL, Tom J and Cooney RV (1993) Inhibitors of endogenous nitrogen oxide formation blocks the promotion of neoplastic transformation in C3H 10T1/2 fibroblasts. *Carcinogenesis* **14**: 1555–1559

Munoz-Fernandez MA, Fernandez MA and Fresno M (1992) Activation of human macrophages for the killing of intracellular *Trypanosoma cruzi* by TNF-α and INF-γ through a nitric oxide-dependent mechanism. *Immunol Lett* **33**: 35–40

Nathan CF and Hibbs JB (1991) Role of nitric oxid synthesis in macrophage antimicrobial activity. *Curr Opin Immunol* **3**: 65–75

Penn I (1986) Cancer is a complication of severe immunosuppression. *Surg Gynecol Obstet* **162**: 603–610

Radomski MW, Jenkins DC, Holmes L and Moncada S (1991) Human colorectal adenocarcinoma cells: differential nitric oxide synthesis determines their ability to aggregate platelets. *Cancer Res* **51**: 6073–6078

Sagar SM, Singh G, Hodson DI and Whitton AC (1995) Nitric oxide and anti-cancer therapy. *Cancer Treat Review* **21**: 159–181

Stuehr DJ and Nathan CF (1989) Nitric oxide. a macrophage product responsible for cytostasis and respiratory inhibition in tumor target cells. *J Exp Med* **169**: 1543–1555

Takeo S, Yasumoto K and Nagashima A (1986) Role of tumor-associated macrophages in lung cancer. *Cancer Res* **46**: 3179–3192

Thomsen LL, Lawton FG, Knowles RG, Beeley JE. Riveros-Moreno V and Moncada S (1994) Nitric oxide synthase activity in human gynecological cancer. *Cancer Res* **54**: 1352–1354

Thomsen LL, Miles DW, Happerfield L, Bobrow LG, Knowles RG and Moncada S (1995) Nitric oxide synthase activity in human breast cancer. *Br J Cancer* **72**: 41–44

Villiotou V and Deliconstantinos G (1995) Nitric oxide, peroxynitrite and nitroso-compounds formation by ultraviolet A (UVA) irradiated human squamous cell carcinoma: potential role of nitric oxide in cancer prognosis. *Anticancer Res* **15**: 931–942

Walters S, Govoni D, Bottazzi B and Mantovani A (1991) The role of macrophages in the regulation of primary tumor growth. *Pathology* **59**: 239–242

WHO (1981) International histologic classification of tumors, no. 1. In *Histologic Typing of Lung Tumors*, 2nd edn, World Health Organization: Geneva

© Cancer Research Campaign 1998